# Master manipulator: small brown planthopper tailors expression of salivary protein to evade plant immunity

**DOI:** 10.1111/tpj.71098

**Published:** 2026-09-09

**Authors:** Brian C. Mooney

The small brown planthopper (SBPH) *Laodelphax striatellus* is a herbivorous insect and highly destructive pest of multiple crops (Li et al., 2023). SBPH feeds on the contents of the phloem as a ‘sap‐sucker’, impeding plant growth and facilitating the spread of viruses. To protect themselves against insect herbivory, plants rely on physical barriers and a range of inducible cellular defence responses. These are triggered by the recognition of insect‐derived components, including proteins secreted in saliva during feeding. For example, perception of the salivary protein Mp10 from the green peach aphid leads to the production of reactive oxygen species (ROS) and the accumulation of defence hormones (Rodriguez et al., 2014). In contrast, salivary proteins may also function as crucial ‘effectors’ that promote herbivory by suppressing defence responses of non‐adapted plant hosts. This results in a trade‐off where the immune‐suppressing benefit of an effector must be weighed against the risk of triggering additional defence responses.

**Figure 1:**
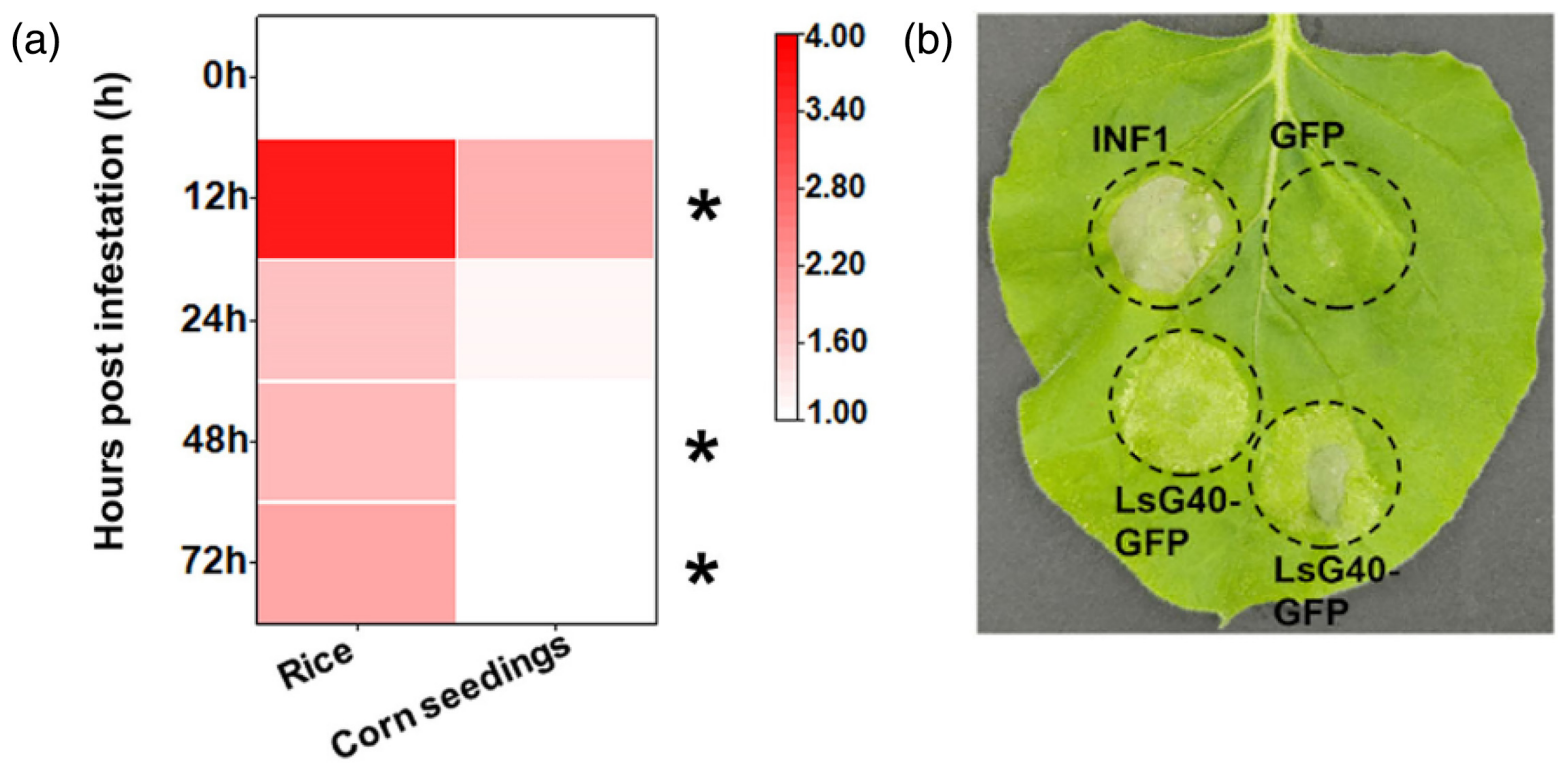
*LsG40* is expressed during herbivory and triggers plant immune responses. (a) Expression of *LsG40* in SBPH after feeding on rice and corn seedlings. Asterisks indicate significant differences between the two hosts. (b) LsG40 induces cell death upon transient expression in *Nicotiana benthamiana*. Expression of GFP and INF1 serve as negative and positive controls, respectively.

Although SBPH can feed on a wide range of plants, rice is its preferred host, providing optimal conditions for maximal survival and fecundity. Maize serves as a suboptimal host that supports feeding but cannot sustain SBPH's full life cycle (Li et al., 2009). Researchers Yongjun Zhang at Nanjing Agricultural University and Zewen Liu at the Institute of Plant Protection in Beijing collaborated in a recent study to investigate the strategies used by SBPH to infest these different plant hosts (Teng et al., 2026). In an initial screen, the authors examined host‐dependent differences in the expression of putative SBPH salivary effectors. While the majority of SBPH genes encoding known salivary proteins did not exhibit host‐dependent differences, the transcript *LsG40* was markedly more abundant in SBPH feeding on the optimal host rice compared with the suboptimal host maize (Figure 1a). This gene is predominantly expressed in the salivary glands and highly enriched in the feeding stages of development compared to the non‐feeding egg stage. Together with the presence of a signal peptide for secretion, this data indicates that *LsG40* encodes a salivary protein with a host‐dependent expression pattern.

To investigate the role of LsG40 *in planta*, an LsG40‐GFP fusion protein was transiently expressed in *Nicotiana benthamiana*. LsG40‐GFP triggered plant defence responses including cell death (Figure 1b) and ROS accumulation, suggesting that this protein is recognised by plant cells as an immune elicitor. These induced defences bolstered plant resistance against the oomycete pathogen *Phytophthora capsici* and rendered plants expressing LsG40 less attractive to herbivory by the fall armyworm (*Spodoptera frugiperda*) compared to a GFP‐expressing control.

The authors next evaluated the effects of LsG40 on SBPH's natural hosts, maize and rice. Application of purified LsG40 protein induced robust defence responses in maize, including callose deposition and transcriptional activation of jasmonic acid pathway‐related genes. Gas chromatography–mass spectrometry (GC–MS) analysis additionally revealed that LsG40‐treated maize plants emitted increased levels of several volatile organic compounds (VOCs) implicated in insect deterrence, including (*E*)‐2‐nonenal and heptadecane (Chamberlain et al., 1991). LsG40 also induced defence responses in the optimal host rice. However, these were generally weaker compared to those of maize, as evidenced by reduced callose deposition and a weaker impact on VOC emissions.

Finally, the authors investigated the effect of *LsG40* silencing on SBPH feeding and fecundity. *LsG40* was knocked down by RNA interference following delivery of dsRNA to SBPH *via* a nanocarrier‐based transdermal system. Depletion of *LsG40* increased the duration of SBPH feeding time on both maize and rice. The number of eggs laid on rice also increased when *LsG40* was silenced. These findings are consistent with the observation that LsG40 is perceived as an immune elicitor in each of these hosts.

In summary, this study identified LsG40 as a novel salivary protein in the small brown planthopper. LsG40 activates defence responses in a variety of plant species, including *N. benthamiana* and natural hosts rice and maize. Despite this, *LsG40* is expressed at a high level when SBPH feeds on rice, its optimal host. This suggests that LsG40 confers a benefit to SBPH fitness when feeding on rice, although the exact nature of this function remains unknown. When SBPH feeds on maize, *LsG40* expression is significantly reduced. The authors propose that *LsG40* levels are controlled to avoid activating defence responses in maize, which are more robust compared to rice. By tailoring expression of *LsG40*, SBPH adapts to enable feeding on a suboptimal host. This study adds a new layer to our understanding of plant–pest interactions, as an example of an insect evading plant immunity by reducing expression of an elicitor rather than suppressing immunity through the activity of an effector (Teng et al., 2026).
